# The prognostic value of mechanical left ventricular dyssynchrony in patients with acute coronary syndrome

**DOI:** 10.1186/1476-7120-11-35

**Published:** 2013-10-11

**Authors:** Carl Westholm, Jonas Johnson, Tomas Jernberg, Reidar Winter

**Affiliations:** 1 Department of Medicine, Section of Cardiology, Huddinge, Karolinska Institutet, Stockholm, Sweden; 2 School of technology and health, Department of Medical Engineering, KTH-Royal Institute of Technology, Stockholm, Sweden; 3 Department of Cardiology, Karolinska University Hospital, Huddinge, Institution of Medicine (H7), Huddinge; Karolinska Institutet, Stockholm 141 86, Sweden

**Keywords:** Acute coronary syndrome, Prognostic parameters, Tissue doppler, Dyssynchrony

## Abstract

**Background:**

Echocardiography is a well-established tool for risk stratification in patients with acute coronary syndrome (ACS). ACS has significant impact on LV dyssynchrony, and detrimental effects on systolic function and long term outcome. The aims of this study were to determine whether LV dyssynchrony carries any predictive information in an unselected ACS population and to evaluate if it has any incremental value to the information given from conventional echocardiographic measurements.

**Methods:**

The study included 227 consecutive ACS patients. Primary endpoint was the composite of death, new MI, or rehospitalisation due to heart failure. Dyssynchrony was measured as intersegmental variation of time to peak strain, the post systolic index (PSI) and myocardial performance index (MPI) with the standard deviation and difference between lowest and highest value (delta) expressing the amount of dyssynchrony. Septal-lateral delay was also tested. All dyssynchrony parameters were compared with Ejection fraction (EF).

**Results:**

The median follow up time was 53 months. 85 patients reached the combined endpoint. Patients with and without a subsequent combined endpoint differed significantly regarding calculated SD: s and delta-value for PSI, time to peak 2D-strain and MPI but not regarding septal-lateral delay. In ROC-analysis none of the dyssynchrony parameters had larger AUC than EF. When adjusting for traditional risk factors none of the dyssynchrony parameters remained associated with outcome, whereas EF still did.

**Conclusion:**

LV dyssynchrony seem to have significant prognostic information in patient with acute coronary syndrome but in comparison to conventional parameters such as EF there is no incremental value of this information.

## Background

Echocardiography is a well-established tool for risk stratification and therapy guidance in patients having acute coronary syndrome (ACS), and echocardiographic measurements such as ejection fraction (EF), wall motion score index (WMSI), the ratio of early mitral inflow over myocardial velocity (E/é), myocardial strain and strain-rate reflecting left ventricular (LV) systolic function and contractility are associated with long-term outcome [1-6].

Regardless of QRS width [7], ACS has significant impact on LV dyssynchrony, and this has been shown to have detrimental effects on the systolic function [8]. Dyssynchrony furthermore predicts LV remodeling [9,10] and, in a population with impaired LV function, also long term outcome [11]. Whether LV mechanical dyssynchrony actually have an incremental prognostic value in unselected patients with ACS in addition to traditional measures of systolic function has not been studied to our knowledge.

Intraventricular dyssynchrony of the LV, i.e. difference in timing of myocardial contraction between the different segments of the LV, can be derived from both tissue Doppler imaging (TDI) and speckle tracking from 2D-images using a wide range of different measures.

The aims of this study were to determine whether LV dyssynchrony carries any predictive information in an unselected ACS population and to evaluate if this prognostic information has any incremental value to the information given from conventional and established echocardiographic measurements.

## Methods

### Study population

The study included 227 patients admitted to the coronary care unit at Karolinska University Hospital, Huddinge, between August 2006 and January 2008, with a clinical diagnosis of ACS. The patients were consecutively included except for temporary interruptions of the study due to high work load at the coronary care unit. All patients underwent clinical assessment including clinical history, physical examination, standard 12-lead ECG, ECG-monitoring and serial measurement of biochemical cardiac markers up to 9–12 hours after admission. All other examinations as well as specific treatments of the patients were left to the discretion of the individual cardiologist. Clinical data were prospectively collected and entered into a database. An acute MI was defined according to current guidelines [12].

The primary endpoint was the composite of death from any cause, new MI, or rehospitalisation due to heart failure. Secondary endpoints were death from any cause, new MI and rehospitalisation due to heart failure as separate endpoints and the composite endpoint of death from any cause or rehospitalisation due to heart failure. All in-hospital events were registered in the study database. Only new MIs occurring more than 24 hours after admission were considered as events. Out of hospital, information about death and need for readmission because of MI or heart failure was obtained by merging the database with the Swedish population registry, which includes information of the vital status of all Swedish citizens, and the National Patient Registry, which includes diagnoses on all patients hospitalized in Sweden.

Before entering the study, all patients gave their written, informed consent. The study was conducted according to the principles of the Declaration of Helsinki and was approved by the local ethics committee.

### Echocardiographic acquisition, analyses and LV dyssynchrony assessment

All echocardiography data were collected at a (median(25th-75th percentile) time of 3(2–4)) days from admission according to the local standard clinical protocol at that time on Karolinska University Hospital by the cardiologist or sonographer on duty that day. The images, including 2D, TDI and spectral Doppler, were collected using a GE Vingmed vivid7 ultrasound machine with standard installed software.

The images were analyzed using a dedicated workstation (EchoPAC, GE Healthcare, Horten Norway) by a well-trained cardiologist (CW) blinded to baseline data and subsequent outcome.

### 2D strain

**Figure 1:**
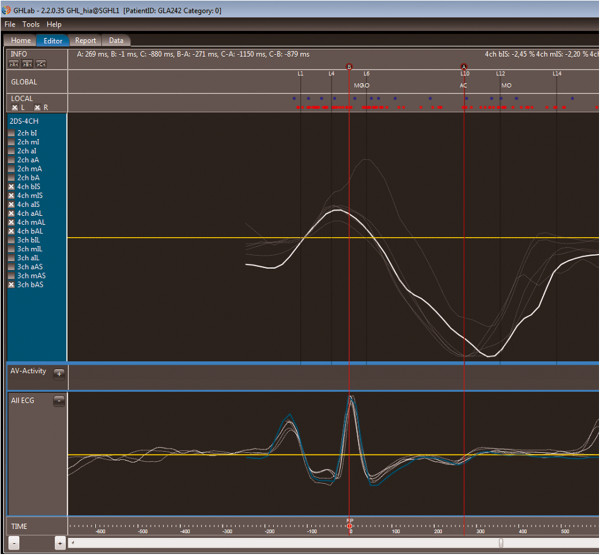
**Assessment of time-to-peak 2D strain.** In the upper half of the figure we see the 2D strain curves for the six segments of the 4-chamber view with the corresponding ECG in the lower half of the figure, one line representing one segment. The left red marker indicates the R-wave from the ECG and the right marker shows the aortic valve closure and end of systole. Time to peak systolic strain is automatically generated for each of these segments and the segments in 2 and 3-chamber view. The Standard deviation of these 18 segments represents Time-to-peak Strain SD, and the difference between the shortest and the longest time Time-to-peak Strain Delta.

Myocardial deformation can be calculated from the 2D images from the *speckle tracking* technique using reflector based identification of the individual reflection pattern of specific bright spots (speckles) in the myocardium and tracking these speckles from frame to frame [13]. With this technique, we can calculate the percentage change in the systolic longitudinal shortening of the myocardium, i.e. longitudinal strain and furthermore the times to peak strain from onset of QRS on a segmental level of the LV. The intersegmental variation of time to peak longitudinal strain measure from speckle tracking has previously been used as a dyssynchrony parameter and has showed to predict outcome after ACS in a population selected for impaired systolic function [11]. This parameter has also been shown to predict the incidence of ventricular arrhythmias [14] which may suggest a prognostic value for CAD patients but has not specifically been tested in an ACS population. The time to peak strain information was exported from GE Echopac and imported to, and processed with GH-lab software (Figure 1) and presented as described below with -SD and –delta parameters. We also present Global Strain which is the average of the segmental peak systolic strain regardless of timing.

The degree of post systolic strain using post systolic index (PSI) also called post systolic shortening (PSS) has been shown to be highly correlated to both chronic and acute ischemia [15] and also a predictor of recovery after NSTEMI [16] but the prognostic value of PSI and it’s intersegmental variation in ACS patients has not been determined. The Post systolic index (PSI) is given automatically in the workstation and is defined as ((peak strain-endsystolic strain)/Peak Strain)×100.

For both PSI and time to peak 2D-strain the intersegmental variation, standard deviation (SD) and the difference between the maximum and minimum (Delta) were used as measurements of LV dyssynchrony.

### Tissue doppler imaging

Measuring the time difference in peak systolic velocity in septum and lateral wall from TDI, often referred to as the *septal-lateral delay*, is both robust and feasible, and one of the most established methods [17]. Also derived from TDI, the ratio between the sum of the isovolumetric relaxation and isovolumetric contraction times devided by contraction time, often referred to as the *myocardial performance index* (MPI) can be calculated, this index is similar to the Tei index, but the latter is not derived from TDI. MPI has shown to predict left ventricular dilatation and death after MI [18] and also reflect the severity of ischemic heart disease [19]. MPI has furthermore been shown to have a prognostic value after ACS in a subgroup of patients with preserved systolic function [20], in patients with ST-elevation myocardial infarction undergoing PCI [21] and also in un unselected population [22]. TDI is a robust measurement and has higher time resolution compared to 2D-imaging which makes it reasonable to assume that the intersegmental variation of MPI could be a suitable way to describe dyssynchrony of the LV. Whether this measurement can be useful as a prognostic marker has not been investigated.

**Figure 2:**
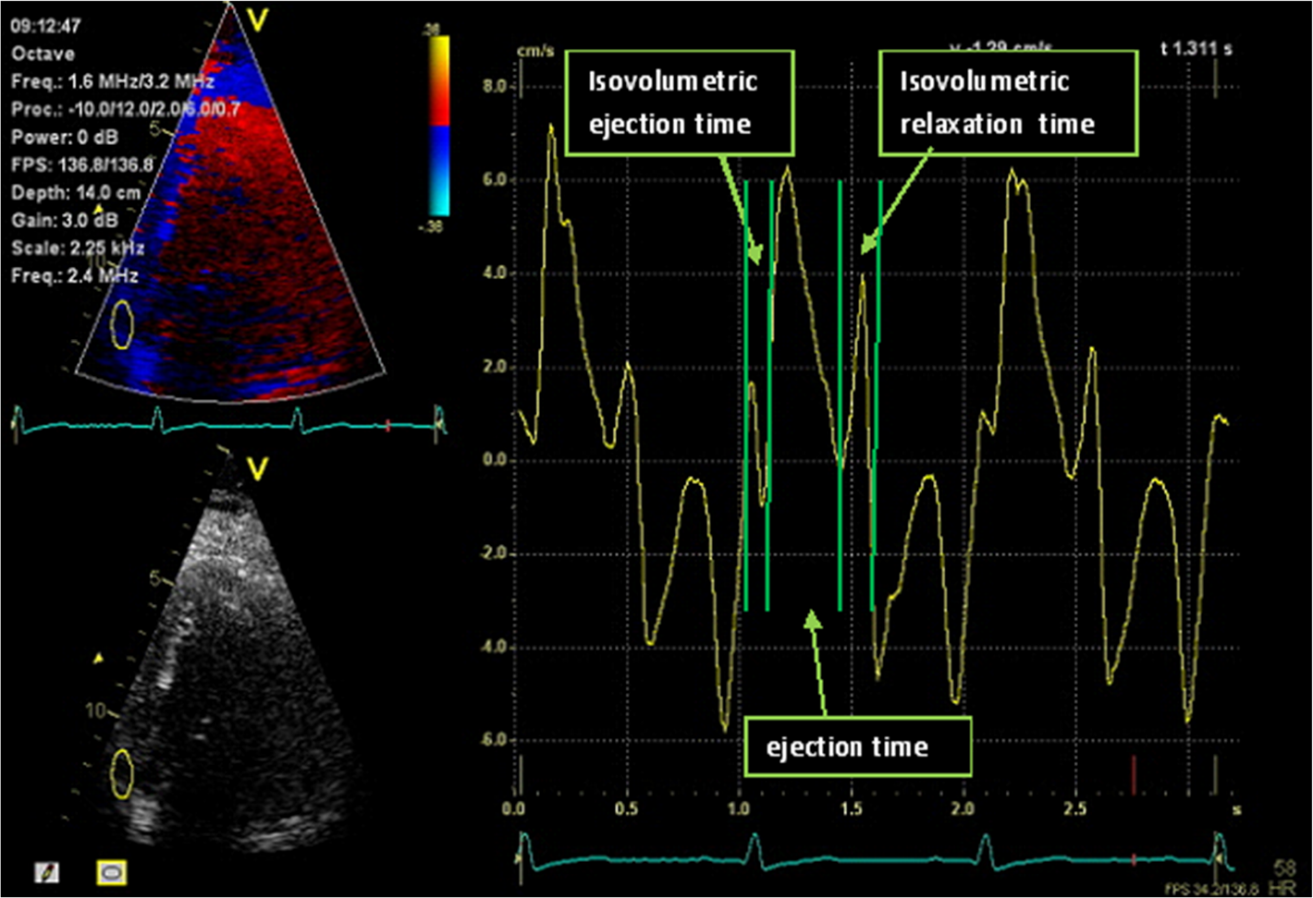
Illustrating how the different time intervals of the MPI are registered from TDI, velocities on the Y-axis and time on the X-axis and under the curve the corresponding ECG.

Both Septal to lateral delay and MPI are acquired in post processing recorded color-coded TDI images using the Q-analysis software in the EchoPAC. Septal-lateral delay is recorded from the four chamber projection as the time difference between peak systolic velocity in the basal segments of the septal and lateral myocardial walls. From the velocity curves and all three apical projections the regional time of the phases of the cardiac cycle where measured and the MPI was calculated, as showed in Figure 2. For each patient MPI was calculated for all six basal segments and SD and delta for MPI as described above.

### Ejection fraction

Ejection fraction was measured according to current EACV/ASE recommendations [23] using the biplane Simpson method of discs from outlining the endocardial border in the apical 4-, and 2-chamber views.

### Wall motion score index

Wall motion score index (WMSI) was derived by a visual assessment of the function of all 18 segments of the left ventricle where normokinetic segments got the value 1, hypokinetic segments 2, akinetic 3 and dyskinetic 4. Wall motion score index was calculated by dividing the sum by 18.

### Statistical analysis

Continuous data are presented as medians with interquartile range (IQR) and categorical data are presented with frequencies and percentages. When analyzing differences between groups the Mann Whitney-test was used for continuous variables and Chi2-test for categorical variables. To compare the prognostic value regardless of chosen cut off-value, receiver operating characteristics (ROC) analyses expressing prognostic value as area under curve (AUC) with 95% confidence interval (CI)were used and the significance of this measurements are evaluated according to Hanley and McNeil [24]. We also included (EF*Time-to-peak Strain SD) and (EF*PSI SD) to examine whether the combination of the two variables could give a larger AUC than when variables were used alone.

To examine whether measurements of dyssynchrony were independently associated with outcome, Cox-regression analyses were used in two models. In model 1 the analysis was made univariate and the parameters were entered one by one without adjustment for other variables. In model 2, adjustment was made for baseline characteristics well known to be associated with outcome (age, gender, diabetes, hypertension, previous heart failure, creatinine clearance and troponin level) and in this model, just as in model 1 the echocardiographic parameters was inserted one by one.

## Results

A total of 227 patients were included in the study. The median follow up time was 53 (range 48–58) months. During this period 85 (37%) patients reached the combined endpoint, among them 42 (19%) died, 48 (21%) had a MI and 52 (23%) were readmitted because of an episode of heart failure. The baseline characteristics in all patients and in those with and without a subsequent combined endpoint are presented in Table 1.

**Table 1:** Baseline characteristics in patients with and without the combined endpoint (n = 227)

	**All (n = 227)**	**No death, MI or HF (n = 142)**	**Death, MI or HF (n = 85)**	
**Variables**	**n (%)**	**n (%)**	**n (%)**	**P**
**Demographics:**				
Age (median, 25th–75th perc.)	67 (59–77)	62 (56–74)	74 (63–80)	<0.001
Men	172 (76)	111 (78)	61 (72)	0.276
**Risk factors:**				
Hypertension	121 (53)	63 (44)	58 (68)	<0.001
Diabetes Mellitus	51 (22)	29 (20)	22 (26)	0.340
Current smoker (missing n = 6)	43 (19)	33 (23)	10 (13)	0.049
**Previous cardiovascular disease:**				
Myocardial infarction	56 (25)	31 (22)	25 (30)	0.200
Heart Failure	19 (8)	2 (1)	17 (20)	<0.001
Revascularization, PCI	33 (15)	23 (16)	10 (12)	0.359
Revascularization, CABG	10 (4)	4 (3)	6 (7)	0.132
Stroke	17 (8)	10 (7)	7 (8)	0.741
**Laboratory measurements**				
NTproBNP 24 h (median, 25th–75th perc.) (n = 189))	1220 (535–3465)	724 (303–1887)	2300 (1030–26040)	<0.001
eGFR (median, 25th–75th perc.) (n = 220)	81 (58–110)	92 (72–116)	66 (40–96)	<0.001
Troponin I (median, 25th–75th perc.) (n = 207)	3.5 (0.41–10.4)	2.4 (0.28–9.6)	4.4 (0.57–10.7)	0.118
**Index Diagnosis**				
Myocardial infarction	188 (83)	119 (84)	69 (81)	0.612
**Measurements of LV function**				
EF simpson (median, 25th–75th perc.)	49 (41–56)	45 (35–52)	52 (45–58)	<0.001
WMSI (median, 25th–75th perc.)	1.06 (1.00–1.33)	1.14 (1.00–1.56)	1.00 (1.00–1.16)	<0.001
**Intervention during admission**				
Coronary angiography	192 (85)	132 (93)	60 (70)	<0.001
PCI	109 (48)	81 (57)	28 (33)	<0.001
CABG	25 (11)	13 (9)	12 (14)	0.248
**Treatment at discharge**				
Betablocker	211 (93)	135 (95)	76 (89)	0.114
Statin	209 (92)	137 (96)	72 (85)	0.003
ASA	217 (96)	141 (99)	76 (89)	0.001
Clopidogrel	148 (65)	103 (73)	45 (53)	0.003
ACE inhibitor/A2 blocker	173 (76)	101 (71)	72 (85)	0.012

### Prognostic value

Patients with and without a subsequent combined endpoint differed significantly regarding both calculated SD: s and delta-value for PSI and for time to peak 2D-strain and MPI but not for septal-lateral delay (Tables 2 and 3). The pattern was similar when patients were divided according to each endpoint separately or to the composite endpoint of death from any cause or rehospitalisation due to heart failure (Table 3).

**Table 2:** All tested echocardiographic parameters in patients with and without the isolated endpoint death and the combined endpoint of death, MI and Readmission due to heart failure

	**Death**			**Composite endpoint death, MI and readmission due to heart failure**		
	**Yes**	**No**	**p**	**Yes**	**No**	**p**
**Dyssynchrony parameters**						
Sept-lat delay (median, 25th–75th perc.)	35 (12.5–105.5)	18 (0–70)	P = 0.060	25 (0–84)	18.5 (0–69.3)	P = 0.387
PSI SD (median, 25th–75th perc.)	20.8 (14.4–28.9)	12.0 (6.1–18.7)	P < 0.001	17.0 (9.3–27.7)	11.0 (6.1–17.8)	P = 0.001
PSI delta (median, 25th–75th perc.)	72 (46.5–103)	36.5 (21–63.7)	P < 0.001	61.5 (29–97.2)	36 (21–62.5)	P = 0.001
Time to peak 2D-strain SD (median, 25th–75th perc.)	0.041 (0.017–0.064)	0.017 (0.007–0.047)	P = 0.003	0.031 (0.016–0.065)	0.015 (0.008–0.045)	P = 0.001
Time to peak 2D-strain delta (median, 25th–75th perc.)	0.13 (0.053–0.23)	0.058 (0.026–0.17)	P = 0.003	0.11 (0.044–0.23)	0.048 (0.026–0.14)	P = 0.001
MPI SD (median, 25th–75th perc.)	0.17 (0.12–0.24)	0.13 (0.09–0.28)	P = 0.003	0.16 (0.11–0.24)	0.13 (0.09–0.18)	P = 0.006
MPI Delta (median, 25th–75th perc.)	0.40 (0.31–0.63)	0.32 (0.22–0.48)	P = 0.005	0.39 (0.27–0.60)	0.31 (0.22–0.45)	P = 0.004
**Non-timing Parameters**						
EF_Simpson (median, 25th–75th perc.)	41.0 (33.0–50.0)	50.0 (44.0–58.0)	P < 0.001	45.0 (35.0–52.0)	52.0 (45.0–58.2)	P < 0.001
Global Strain (median, 25th–75th perc.)	-10.3 (-12.5–7.6)	-14.4 (-16.4–11.9)	P < 0.001	-11.2 (-14.5–7.8)	-14.6 (-16.7–12.3)	P < 0.001
WMSI (median, 25th–75th perc.)	1.33 (1.0–1.63)	1.0 (1.9–1.2)	P < 0.001	1.13 (1.0–1.56)	1.00 (1.00–1.11)	P < 0.001
PSI (median, 25th–75th perc.)	13.7 (8.7–22.1)	7.6 (3.1–13.7)	P < 0.001	11.3 (5.1–18.7)	7.6 (3.3–13.4)	P = 0.007

(n = 227).

**Table 3:** All tested echocardiographic parameters in patients with and without the isolated endpoint MI and the combined endpoint of death and readmission due to heart failure

	**MI**			**Composite endpoint, Death and readmission due to heart failure**		
	**Yes**	**No**	**p**	**Yes**	**No**	**p**
**Dyssynchrony parameters**						
Sept-lat delay (median, 25th–75th perc.)	28.0 (0.0–73.8)	20.0 (0.00–111.2)	P = 0.629	28 (0.00–94.0)	18.0 (0.00–107.2)	P = 111
PSI SD (median, 25th–75th perc.)	16.2 (9.7–30.1)	12.5 (6.1–20.3)	P = 0.009	19.9 (11.5–28.7)	12.4 (6.7–18.23)	P < 0.001
PSI delta (median, 25th–75th perc.)	55.5 (34.3–102.3)	41 (21–67)	P = 0.019	66.0 (32.0–103.0)	41.0 (22.0–53.5)	P < 0.001
Time to peak 2D-strain SD (median, 25th–75th perc.)	0.032 (0.016–0.068)	0.017 (0.009–0.047)	P = 0.019	0.041 (0.17–0.66)	0.14 (0.007–0.032)	P < 0.001
Time to peak 2D-strain delta (median, 25th–75th perc.)	0.10 (0.050–0.23)	0.057 (0.026–0,17)	P = 0.030	0.14 (0.049–0.24)	0.044 (0.023–0.116)	P < 0.001
MPI SD (median, 25th–75th perc.)	0.16 (0.09–0.24)	0.14 (0.090–0.20)	P = 0.117	0.17 (0.12–0.24)	0.13 (0.088–0.19)	P = 0.001
MPI Delta (median, 25th–75th perc.)	0.41 (0.24–0.58)	0.33 (0.23–0.50)	P = 0.74	0.42)0.32–0.60)	0.33 (0.22–0.48)	P = 0.001
**Non-timing Parameters**						
EF_Simpson (median, 25th–75th perc.)	44 (35–52)	51 (43–58)	P = 0.002	41 (33.7–51.0)	52.0 (45.5–63.2)	P < 0.001
Global Strain (median, 25th–75th perc.)	-10.9 (-14.6–7.7)	-14.4 (-16.6–11.4)	P = 0.002	-10.6 (-14.3–7.6)	-14.7 (-16.9–12.3)	P < 0.001
WMSI (median, 25th–75th perc.)	1.22 (1.0–1.54)	1.00 (1.00–1.16)	P = 0.026	1.27 (1.00–1.67)	1.00 (1.00–1.50)	P < 0.001
PSI (median, 25th–75th perc.)	10.3 (7.00–24.7)	8.7 (3.9–15–3)	P = 0.058	12.7 (6.6–22.9)	8.5 (3.8–13.6)	P = 0.004

(n = 227).

When the associations between tested parameters and outcome were evaluated with ROC-analyses, dyssynchrony parameters based on PSI and time to peak 2D-strain were more associated with the composite endpoint than both MPI and septal-lateral delay but none of the dyssynchrony parameters had a larger AUC than EF according to Simpson regardless of endpoint (Table 4). None of the differences were statistically significant. The combination of EF and the dyssynchrony parameters Time-to-peak Strain SD and PSI SD did not generate higher AUC: s than either of the parameters isolated ((EF*Time-to-peak Strain SD: 0.61) and (EF*PSI SD: 0.57)).

**Table 4:** ROC-analysis with AUC for all the echocardiographic measurements in respect to death and the two different combined endpoints

	**AUC (95%CI)**	**AUC (95%CI)**	**AUC (95%CI)**
	**Death**	**Composite endpoint, death, heart failure and MI**	**Composite endpoint, death and heart failure**
	**n = 42**	**n = 85**	**n = 68**
**Dyssynchrony parameters**			
Sept-lat delay	0.59 (0.49–0.79)	0.53 (0.45–0.62)	0.57 (0.48–0.66)
PSI SD	0.72 (0.64–0.80)	0.64 (0.56–0.72)	0.67 (0.59–0.75)
PSI delta	0.72 (0.63–0.80)	0.64 (0.56–0.72)	0.66 (0.58–0.75)
Time to peak 2D-strain-SD	0.65 (0.56–0.74)	0.64 (0.56–0.72)	0.68 (0.59–0.76)
Time to peak 2D-strain-delta	0.65 (0.56–0.74)	0.64 (0.56–0.72)	0.69 (0.60–0.77)
MPI SD	0.65 (0.56–0.74)	0.61 (0.53–0.68)	0.64 (0.56–0.72)
MPI Delta	0.64 (0.55–0.73)	0.61 (0.54–0.69)	0.64 (0.56–0.72)
**Non-timing parameters**			
Simpson EF	0.73 (0.65–0.81)	0.68 (0.60–0.76)	0.71 (0.63–0.79)
Global Strain	0.76 (0.68–0.84)	0.72 (0.64–0.79)	0.74 (0.67–0.82)
WMSI	0.68 (0.58–0.77)	0.65 (0.58–0.73)	0.69 (0.61–0.77)
PSI	0.68 (0.60–0.77)	0.60 (0.51–0.67)	0.61 (0.53–0.70)

In a Cox regression analysis when adjusting for traditional risk factors (age, gender, diabetes, hypertension, previous heart failure, creatinine clearance and troponin levels) none of the dyssynchrony parameters remained independently associated with outcome, whereas EF according to Simpson still did (Table 5).

**Table 5:** Cox regression analyses

	**Cox regression analysis**	**Cox regression analysis**
	**Model 1 univariate**	**Model 2**
	**HR (95% CI)**	**HR (95% CI)**
Sept-lat delay	1.002 (0.997–1.006)	0.999 (0.987–1.008)
PSI SD	1.017 (1.005–1.029)	1,007 (0.989–1–026)
PSI delta	1.004 (1.001–1.008)	1.002 (0.996–1.007)
Time to peak 2D-strain SD ×100	1.088 (1.027–1.151)	1.016 (0.934–1.106)
Time to peak 2D-strain delta	31.81 (4.267–237.1)	2.345 (0.160–34.45)
MPI SD	1.798 (0.602–5.370)	1.076 (0.240–4.819)
MPI Delta	1.291 (0.831–2.007)	1.004 (0.569–1–016)
Simpson EF	0.947 (0.947–0.979)	0.977 (0.955–0.999)

In model 1 no adjustment was made. In Model 2 adjustment was made for age, gender, diabetes, hypertension, heart failure, creatinine clearance and troponin levels. The echocardiographic parameters are tested one by one with the risk factors and not at the same time.

## Discussion

The main findings of this study are that the dyssynchrony parameters do carry prognostic information regarding long term outcome after ACS in an unselected population. However, the clinical value of this information is limited since the dyssynchrony parameters seem to have only a moderate predictive prognostic value and no incremental value to well known risk factors such as age, gender, diabetes, hypertension, previous heart failure, creatinine clearance and troponin levels and no incremental value to EF, the most established and commonly used method for assessment of systolic LV function. Given the previous data of the importance of LV dyssynchrony in various clinical patients groups, the seemingly relatively weak prognostic value of LV dyssynchrony in this setting of unselected ACS patients is both somewhat surprising and not easily understandable.

One possible explanation might be that these parameters, which have consistently been shown to be very sensitive to detect ischemia in previous studies [7,9-11,25], could therefore be significantly deranged already at relatively small ischemic burden and not increase proportionally with increasing ischemic burden. The dyssynchrony could thus be relatively similar in the whole ACS population compared to the normal population and accordingly not reflect the actual size of the damaged myocardium. The dyssynchrony might therefore not be strongly associated with the subsequent loss of contractility and heart failure in long-term outcome.

Furthermore there is a lack of detailed knowledge about the time course of the different timing disturbances after ACS. It might therefore be possible that myocardial contractility in a reverse remodeling context actually recover earlier than LV dyssynchrony and if this is true, dyssynchrony measured in the early phase in the recovering myocardium might give a false measure of risk. The result might in fact have been different if the images had been acquired earlier or later after the ACS episode, for example within the first hours of admission or 3–4 weeks.

Another possibility is following from the fact that dyssynchrony parameters reflect the relative intersegmental differences. We need to keep in mind that a very large myocardial infarct will affect a larger number of LV segments, which over a certain level could lead to a seemingly paradoxically lower intersegmental variation, Thus, a large myocardial infarction does not necessarily result in a high degree of dyssynchrony.

Post systolic contraction, measured with PSI, is known to be highly correlated to ischemia [15] and seem to have a higher predictive value in the present study compared to the other, more electromechanical parameters. This observation suggests that it is the remaining ischemia at the time of examination might be the main determinant of worse prognosis rather than LV dyssynchrony.

The fact that some echocardiographic parameters in this study can predict all the endpoints, both isolated and combined, and with or without new MI indicates that there are unknown confounding factors behind both severity of coronary disease and disturbance of LV function of clinical relevance even though, intuitively, it is hard to see the direct correlation between a plaque rupture and LV dysfunction.

One interesting finding, however, is the fact that the predictive value of these parameters seem to be better using death as endpoint compared to the other endpoints. The small number of events and the lack of significant results in this study allow for nothing more than speculations. But nevertheless, one possible explanation might be that death in this group to some extent is due to ventricular arrhythmias and that the dyssynchrony parameters actually predict that more than heart failure and new ischemic events. This is to some extent supported in earlier studies, even though not in an ACS population [14].

### Limitations

A major limitation of this study is that we did not have a standardized time between the coronary angiography and the echocardiographic examination in relation to time of admission. If all echocardiographic examinations would have been performed within the first 24 hours after diagnose and prior to angiography the result might have been different. The result might also have been different if we had included a 3–6 months follow up echocardiographic examination to assess the amount of reverse remodeling and actual persisting damage.

Another fact that could be considered as a limitation is the image quality. All the images used in this study were stored from everyday clinical examinations and not by a highly specialized research lab and it is reasonable to believe that the image quality is more essential for timing parameters compared to conventional parameters, which might have affected the results. On the other hand this is also one of the strengths of this study, pointing out the limitation of using the dyssynchrony parameters with the technique and resources we have at hand today in the clinical daily routine.

## Conclusion

Mechanical LV dyssynchrony seem to carry some significant prognostic information in patient with acute coronary syndrome but in comparison to well-known risk factors and conventional parameters such as EF there is little or no incremental value of this information.

## Competing interests

The authors declare that they have no competing interests.

## Authors’ contributions

All authors have contributed to the manuscript and approved the final version.
